# Promoting the Photoelectrochemical Properties of BiVO_4_ Photoanode via Dual Modification with CdS Nanoparticles and NiFe-LDH Nanosheets

**DOI:** 10.3390/nano14131100

**Published:** 2024-06-26

**Authors:** Guofa Dong, Tingting Chen, Fangxia Kou, Fengyan Xie, Caihong Xiao, Jiaqi Liang, Chenfang Lou, Jiandong Zhuang, Shaowu Du

**Affiliations:** 1 Fuzhou Institute of Oceanography, College of Materials and Chemical Engineering, Minjiang University, Fuzhou 350108, China; gfdong@mju.edu.cn (G.D.);; 2 College of Materials Engineering, Fujian Agriculture and Forestry University, Fuzhou 350002, China

**Keywords:** photoelectrochemical, water splitting, BiVO_4_, CdS, NiFe-LDH, photoanode

## Abstract

Bismuth vanadate (BiVO_4_) has long been considered a promising photoanode material for photoelectrochemical (PEC) water splitting. Despite its potential, significant challenges such as slow surface water evolution reaction (OER) kinetics, poor carrier mobility, and rapid charge recombination limit its application. To address these issues, a triadic photoanode has been fabricated by sequentially depositing CdS nanoparticles and NiFe-layered double hydroxide (NiFe-LDH) nanosheets onto BiVO_4_, creating a NiFe-LDH/CdS/BiVO_4_ composite. This newly engineered photoanode demonstrates a photocurrent density of 3.1 mA cm^−2^ at 1.23 V vs. RHE in 0.1 M KOH under AM 1.5 G illumination, outperforming the singular BiVO_4_ photoanode by a factor of 5.8 and the binary CdS/BiVO_4_ and NiFe-LDH/BiVO_4_ photoanodes by factors of 4.9 and 4.3, respectively. Furthermore, it exhibits significantly higher applied bias photon-to-current efficiency (ABPE) and incident photon-to-current efficiency (ICPE) compared to pristine BiVO_4_ and its binary counterparts. This enhancement in PEC performance is ascribed to the formation of a CdS/BiVO_4_ heterojunction and the presence of a NiFe-LDH OER co-catalyst, which synergistically facilitate charge separation and transfer efficiencies. The findings suggest that dual modification of BiVO_4_ with CdS and NiFe-LDH is a promising approach to enhance the efficiency of photoanodes for PEC water splitting.

## 1. Introduction

The increasing depletion of fossil fuels, environmental degradation, and the threat of global warming represent significant challenges that will persist without intervention. Addressing these issues requires the exploration of alternative energy resources such as wind, solar, hydropower, and sea wave energy [1]. Among these, hydrogen gas is distinguished by its numerous advantages over traditional fuels and is considered the most promising alternative for next-generation energy resources [2]. Hydrogen offers renewable and sustainable energy with the highest known gravimetric energy density, coupled with zero emissions and excellent storability. Therefore, developing environmentally and economically sustainable techniques for hydrogen production is of paramount importance. 

Photoelectrochemical (PEC) water splitting driven by direct sunlight irradiation has been recognized as one of the most promising approaches to generating hydrogen in a green and pollution-free way [3]. This solar-to-hydrogen (STH) energy conversion is achievable through the integration of light-harvesting systems with catalysts that facilitate water splitting. In such devices, metal oxide semiconductors frequently serve as light-absorbing photoelectrodes [4], with BiVO_4_ emerging as one of the most ideal options owing to its narrow bandgap energy of 2.4 eV, appropriate valence-band position for water oxidation, and high STH conversion efficiency of about 9% [5,6,7]. However, the practical use of BiVO_4_ is hampered by several challenges, including slow kinetics of the surface water oxidation reaction (OER), limited carrier mobility, and rapid charge recombination [8,9,10,11], resulting in a STH efficiency considerably lower than the theoretical maximum and the 10% threshold required for practical applications [12,13,14,15,16,17]. Enhancing PEC performance can be pursued by modifying BiVO_4_ photoanodes with OER co-catalysts, such as noble metal oxides including RhO_2_, IrO_2_, and RuO_2_ [18,19], cobalt-phosphate (Co-Pi) [20,21,22,23], and group VIII metal oxides or (oxy)hydroxides [24,25,26,27,28]. These modifications aim to inhibit charge recombination and accelerate the OER rate. Additionally, coupling BiVO_4_ photoanodes with other metal oxide semiconductors, for instance, ZnO, WO_3_, and SnO_2_, or the combination thereof to create heterojunctions, can further improve charge separation and transfer, thereby bolstering PEC performance [29,30,31,32,33,34,35,36]. Despite these advances, the intricate and often expensive process of constructing these integrated BiVO_4_ photoanodes may render them unfeasible for real applications.

Layered double hydroxides (LDHs) represent a versatile family of crystalline materials, notable for their diverse chemical compositions, morphologies, and particle sizes. They are also inexpensive, easy to make, and structurally tunable [37]. Furthermore, LDHs are recognized as one of the most efficient classes of OER catalysts. Certain LDHs have been successfully deposited on BiVO_4_ photoanodes as OER co-catalysts to improve PEC water splitting performance. For instance, integrating NiCo- or NiFe-LDHs with BiVO_4_ photoanodes has been demonstrated to significantly increase the photocurrent density, surpassing that of the unmodified BiVO_4_ photoanode by several folds [38,39,40,41]. Meanwhile, constructing heterojunctions between CdS and BiVO_4_ semiconductors has been effective in improving the separation and transfer efficiencies of photogenerated charge carriers [42,43,44]. However, studies on PEC water splitting using a BiVO_4_ photoanode co-modified with CdS and NiFe-LDH have not been documented. In this study, a triadic photoanode of NiFe-LDH/CdS/BiVO_4_ was facilely fabricated through hydrothermal growth of CdS nanoparticles on the BiVO_4_ surface, followed by electrochemical deposition of NiFe-LDH nanosheets. The resultant photoanode shows good PEC performance with a photocurrent density of 3.1 mA cm^−2^ in 0.1 M KOH at 1.23 V, which is 5.8 times greater than the unmodified BiVO_4_ and 4.9 and 4.3 times greater, respectively, than the binary photoanodes CdS/BiVO_4_ and NiFe-LDH/BiVO_4_. In addition, it also exhibits good stability in photocurrent density under continuous illumination for 3 h.

## 2. Materials and Methods

### 2.1. Materials

Unless otherwise specified, the reagents used in the experiments were analytically pure and were utilized as received without further purification. Deionized water was used throughout all experiments.

### 2.2. Preparation of Photoanodes 

The fluoride–tin oxide (FTO) substrates (30 × 10 × 1.1 mm) were sequentially cleaned via ultrasonication in a 5% glass washing solution, water, acetone, and ethanol for 30 min, followed by air-drying for subsequent utilization. The BiVO_4_ film was synthesized through a two-step synthetic procedure, according to the literature [45]. Initially, 40 mmol of Bi(NO_3_)_3_·5H_2_O was dissolved in 50 mL of HNO_3_ aqueous solution (pH = 1.7). Subsequently, 400 mmol of KI was added and stirred until fully dissolved. To this solution, 20 mL of an ethanol solution containing 230 mmol of p-benzoquinone was added, followed by continuous stirring for an additional 15 min to generate the BiOI precursor solution. Electrodeposition of the BiOI layer on the FTO substrate was performed potentiostatically at −0.1 V versus Ag/AgCl for 180 s at room temperature using a typical three-electrode system: the FTO substrate as the working electrode, a saturated Ag/AgCl as the reference electrode, and a Pt net as the counter electrode. Following electrodeposition, the BiOI electrode was extensively rinsed with water and air-dried at room temperature. Conversion of BiOI to BiVO_4_ involved the application of 70 μL of DMSO solution containing 0.23 mmol of VO(acac)_2_ onto the BiOI electrode (1 × 1 cm), followed by annealing at 450 °C for 2 h (heating rate = 2 °C/min). Residual V_2_O_5_ on the BiVO_4_ surface was removed by immersion in 1 M NaOH solution for 30 min. The final BiVO_4_ photoanode was then rinsed with water and dried at room temperature. 

A solution comprising Cd(NO_3_)_2_·4H_2_O (0.16 mmol), trisodium citrate (0.20 mmol), and thiourea (0.13 mmol) was prepared by dissolving these reagents in 50 mL of distilled water, followed by sonication for 10 min. The pH of the solution was adjusted to 11 by the gradual addition of ammonia solution until a yellow precipitate formed. Subsequently, a BiVO_4_ photoanode, suspended from an iron wire, was immersed vertically in this suspension. The solution was then heated at 90 °C in an oil bath for 30 min. After that, the BiVO_4_ photoanode was removed and washed repeatedly with water to yield the CdS/BiVO_4_ photoanode. This photoanode was further processed by immersion in a mixed solution of 0.1 M Ni(NO_3_)_2_·6H_2_O and 0.1 M FeSO_4_·7H_2_O. Potentiostatic deposition was executed at −1 V for 180 s. Following this, the photoanode was thoroughly rinsed with water and dried at 60 °C for 2 h in an oven to complete the fabrication of the NiFe-LDH/CdS/BiVO_4_ photoanode.

### 2.3. Measurements

The surface morphology of the samples was analyzed using a Hitachi SU8000 field emission scanning electron microscope (Hitachi, Japan), equipped with energy dispersive X-ray (EDS) and mapping capabilities. High-resolution imaging was conducted using a JEOL JEM-2100 transmission electron microscope (JEOL, Tokyo, Japan). The crystalline phases of the samples were determined through X-ray powder diffraction (XRD) using a Mini FLEX600 (Rigaku, Tokyo, Japan) with Cu Kα radiation (λ = 0.154 nm). Surface chemical compositions were analyzed by X-ray photoelectron spectroscopy (XPS) on a SPECS system operated at 150 W utilizing Al Kα radiation as the X-ray source.

### 2.4. Photoelectrochemical Analysis

Photoelectrochemical measurements were performed at room temperature using an electrochemical workstation CHI 660E (CH Instruments, Shanghai, China) in a three-electrode system. The as-prepared photoanodes, a saturated Ag/AgCl, and a Pt wire mesh were configured as working, reference, and counter electrodes, respectively. A 300 W xenon lamp PLS-FX300HU (PerfectLight, Beijing, China) coupled with an AM 1.5 G filter was used as the light source, and the light intensity was adjusted to 100 mW cm^−2^. All as-prepared photoanodes were illuminated from the back (the irradiated area was 1.0 cm^2^) and were immersed in an aqueous solution of 0.5 M Na_2_SO_4_ as the electrolyte. Potentials reported here, unless otherwise specified, were converted to RHE using Equation (1):

(1) E(vs.RHE)=E(vs. Ag/AgCl)+0.197 V+0.059 V×pH

The photocurrents were measured by linear scanning voltammetry (LSV) with a scanning rate of 10 mV s^−1^ from –0.4 V to 1.2 V. For electrochemical impedance spectroscopy (EIS) measurement, a sinusoidal voltage pulse of 10 mV amplitude was applied to a bias voltage of 0.67 V with frequencies ranging from 100 kHz to 10 mHz. The charge injection efficiencies (η_inj_) and charge separation efficiencies (η_sep_) can be calculated from Equations (2) and (3):

(2) $$\eta_{inj}=\frac{J_{H_{2}O}}{J_{{Na}_{2}{SO}_{3}}}\times 100\%$$

(3) $$\eta_{sep}=\frac{J_{{Na}_{2}{SO}_{3}}}{J_{abs}}\times 100\%$$

where $J_{H_{2}O}$ and $J_{{Na}_{2}{SO}_{3}}$ are the photocurrent densities without and with Na_2_SO_3_ as a hole scavenger, respectively, and J_abs_ is the photocurrent density by assuming 100% absorbed photon-to-current efficiency. The applied bias photon-to-current efficiency (ABPE) was calculated according to Equation (4):

(4) $$ABPE=\frac{J_{ph}\times \left(1.23-V_{app}\right)}{P_{light}}\times 100\%$$

where J_ph_ is the photocurrent density, V_app_ is the applied external potential, and P_light_ is the light density of the illumination (100 mW cm^−2^). The incident photon-to-current conversion efficiency (IPCE) was measured from 350 nm to 650 nm using a monochromator (PLS MC150), which is calculated from Equation (5):

(5) $$IPCE=\frac{\left(J_{light}-J_{dark}\right)\times 1240}{P_{mono}\times \lambda }\times 100\%$$

where J represents the current density under light and darkness, P_mono_ refers to the light intensity, and λ is the incident light wavelength. Mott−Schottky (M−S) spectra were measured in a 0.5 M Na_2_SO_4_ (pH = 6.1) in the dark from 0 V to 0.4 V. The donor density (N_D_) is calculated from Equation (6):

(6) $$\frac{1}{C^{2}}=\frac{2}{e\varepsilon_{0}\varepsilon N_{D}}[\left(E-E_{fb}\right)-kT/e)]$$

where C represents the capacitance of the space charge region, ε_0_ is the vacuum permittivity, ε is the relative permittivity of the semiconductor, e is the electron charge, E is the applied potential, E_fb_ is the flat band potential, k is the Boltzmann constant, and T is the absolute temperature.

A stability test of the photoanodes was conducted under successive illumination for 3 h at 1.23 V. The evolved H_2_ and O_2_ gases were collected and tested in a three-electrode system by a gas chromatograph spectrometer (GC9790II) with a thermal conductivity detector (TCD). The electrolyte was purged with Ar for 30 min to eliminate any dissolved oxygen before the measurement.

## 3. Results

### 3.1. Characterization of the Photoanode Materials

**Figure 1:**
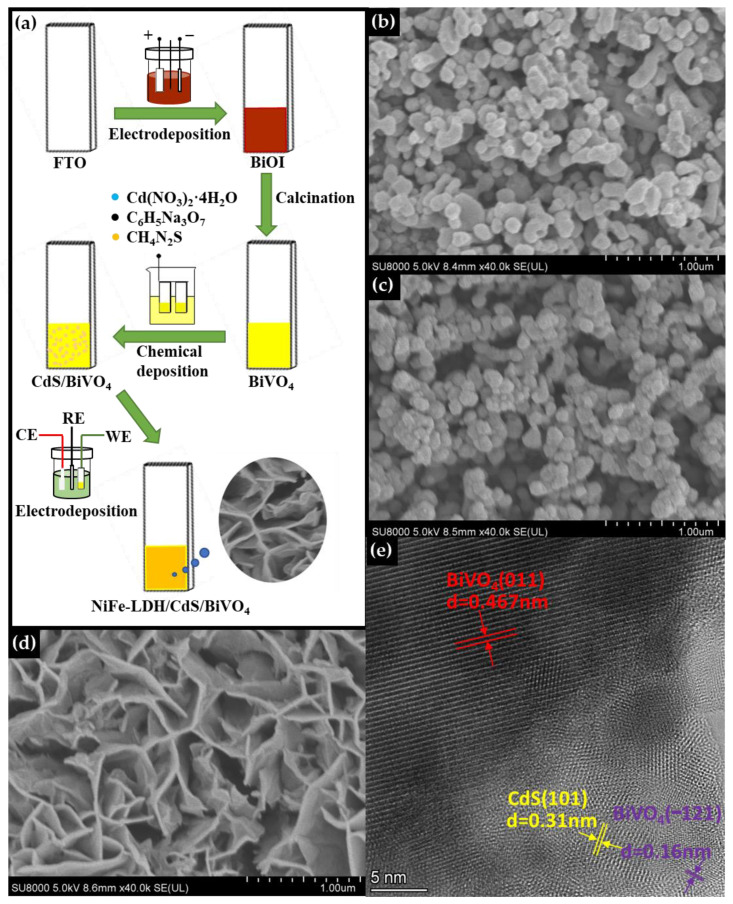
(**a**) The synthetic procedure of the NiFe-LDH/CdS/BiVO_4_ photoanode; SEM images of (**b**) BiVO_4_, (**c**) CdS/BiVO_4_, and (**d**) NiFe-LDH/CdS/BiVO_4_; (**e**) HRSEM image of NiFe-LDH/CdS/BiVO_4_.

The synthetic route for the preparation of the NiFe-LDH/CdS/BiVO_4_ photoanode is presented in Figure 1a. BiVO_4_ was synthesized using a two-step method [45]. First, a precursor solution containing the tetraiodo bismuthate complex [BiI_4_]^–^ was prepared by reacting Bi(NO_3_)_3_·5H_2_O with excess KI under acidic conditions. This solution was then subjected to electrodeposition. During this process, benzoquinone, pre-added to the precursor solution, was electrochemically reduced to hydroquinone at the FTO working electrode. This reduction consumed protons and generated hydroxyl ions at the electrode, which subsequently reacted with [BiI_4_]^–^ to form BiOI on the FTO substrate. In the second step, the BiOI film was converted to BiVO_4_ by annealing at 450 °C in the presence of VO(acac)_2_, likely involving a solid-state reaction between Bi_2_O_3_ and V_2_O_5_. Next, CdS nanoparticles were grown by reacting Cd^2+^ ions with thiourea in the presence of trisodium citrate at 90 °C. These nanoparticles were then directly deposited onto the BiVO_4_ film to establish the CdS/BiVO_4_ photoanode, which was subsequently coated with ultrathin NiFe-LDH nanosheets by electrochemical deposition. For comparison purposes, the binary counterparts, CdS/BiVO_4_ and NiFe-LDH/BiVO_4,_ were also prepared under similar conditions. The surface morphology and elemental distribution of the photoanode materials were thoroughly characterized using SEM and HRTEM, complemented by EDS mapping. The electrodeposited BiOI exhibited a two-dimensional plate structure oriented perpendicular to the FTO substrate (Figure S1a). Subsequent calcination transformed the BiOI into a BiVO_4_ film, which displayed an irregular, worm-like porous architecture (Figure 1b). The CdS/BiVO_4_ heterojunction maintained a morphology akin to that of BiVO_4_, but with noticeable coverage by small CdS nanoparticles (Figure 1c and Figure S1b). As can be seen from Figure 1d, NiFe-LDH nanosheets were uniformly electrodeposited over the surface of the CdS/BiVO_4_ photoanode. HRTEM analysis, detailed in Figure 1e, identified lattice spacings of 0.16 nm and 0.467 nm, corresponding to the (–121) and (011) crystal planes of BiVO_4_, respectively. Additionally, a lattice fringe of 0.31 nm was observed, indicative of the (101) plane of CdS, confirming the successful formation of the CdS/BiVO_4_ heterojunction. SEM-EDS mapping verified the homogeneous presence of Bi, V, O, Cd, S, Ni, and Fe elements (Table S1), demonstrating successful loading of CdS and NiFe-LDH on the BiVO_4_ photoanode (Figure S2). 

**Figure 2:**
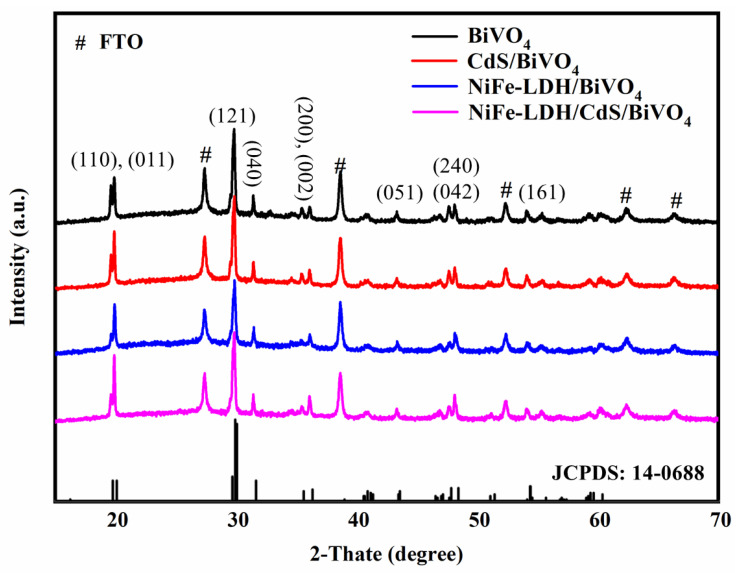
XRD patterns of the photoanode materials.

The crystal phase and structure of the photoanode materials were examined using X-ray diffraction (XRD), with results presented in Figure 2. In addition to the distinct diffraction peaks corresponding to SnO_2_ from the FTO substrate, the XRD spectrum of the as-prepared BiVO_4_ aligns well with the monoclinic scheelite BiVO_4_ (JCPDS: 14-0688). However, no characteristic diffraction peaks for CdS and NiFe-LDH were observed in the XRD spectra of the modified photoanodes. This absence is likely due to the low loading of CdS and the amorphous nature of NiFe-LDH, as corroborated by SEM and HRTEM results. The amorphous structure of the NiFe-LDH nanosheets may introduce abundant defects or vacancies, thereby increasing the active sites on the catalyst surface and facilitating the oxygen evolution reaction (OER) [46].

**Figure 3:**
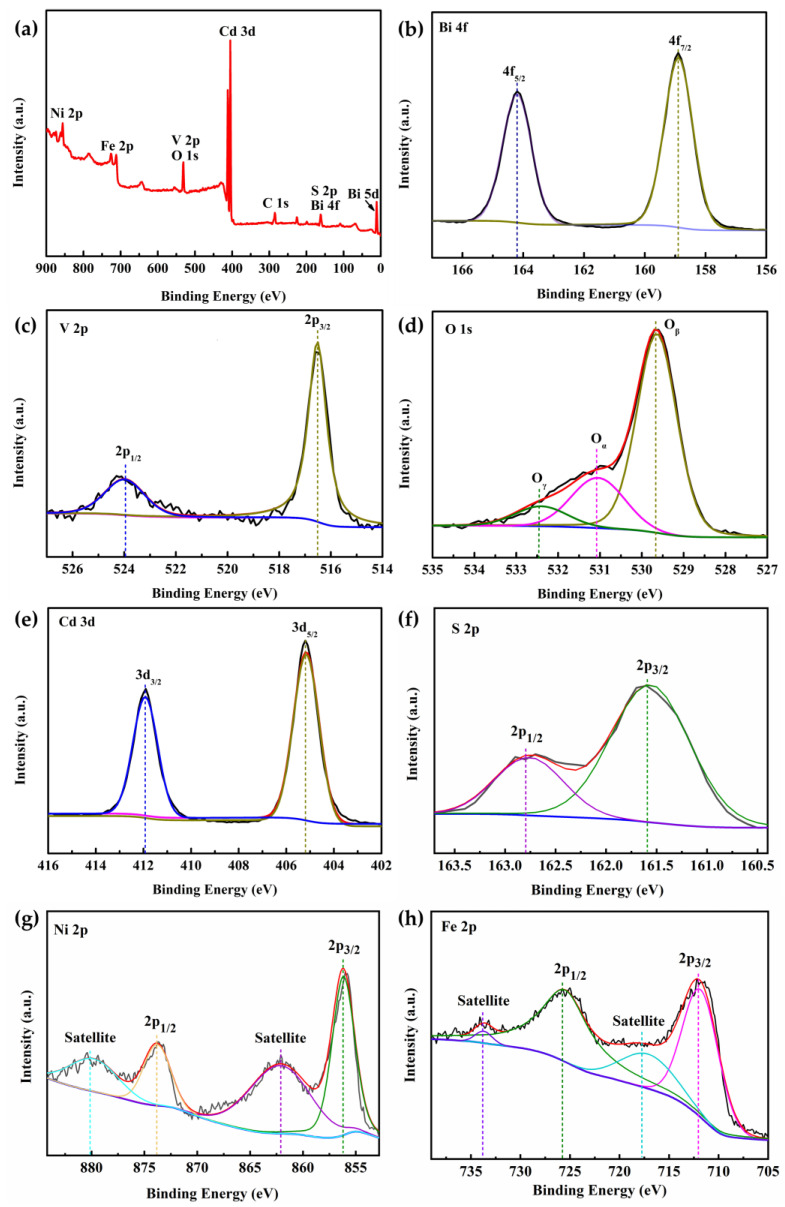
XPS spectra of (**a**) NiFe-LDH/CdS/BiVO_4_, (**b**) Bi 4f, (**c**) V 2p, (**d**) O 1s, (**e**) Cd 3d, (**f**) S 2p, (**g**) Ni 2p, and (**h**) Fe 2p.

The chemical composition and valence states of the NiFe-LDH/CdS/BiVO_4_ photoanode were characterized using XPS. The survey spectrum, depicted in Figure 3a, reveals peaks corresponding to Bi, V, O, Cd, S, Ni, and Fe elements, verifying the formation of the CdS and NiFe-LDH composite. The Bi 4f XPS spectrum (Figure 3b) displays two distinct peaks at 158.89 eV for Bi 4f_7/2_ and 164.2 eV for Bi 4f_5/2_, consistent with the Bi^3+^ state. In Figure 3c, the split peaks of V 2p corresponding to V 2p_3/2_ and V 2p_1/2_ of V^5+^ are found at 516.52 eV and 523.98 eV [47,48]. The O 1s spectrum in Figure 3d features three peaks: lattice oxygen (O_β_) at 529.64 eV, surface adsorbed oxygen species (O_α_) at 531.05 eV, and adsorbed molecular water (O_γ_) at 532.4 eV. In the Cd 3d XPS spectrum (Figure 3e), there are two narrow peaks at 405.16 eV and 411.92 eV, which can be assigned to the 3d_5/2_ and 3d_3/2_ of Cd^2+^. The peaks of the S 2p_3/2_ and S 2p_1/2_ orbitals are located at 161.56 eV and 162.76 eV, respectively, indicating the existence of S^2−^ (Figure 3f) [49]. The Ni 2p spectrum is composed of two major peaks located at 856.19 eV and 873.80 eV, with satellite peaks at 862.17 eV and 879.81 eV (corresponding to Ni 2p_3/2_ and Ni 2p_1/2_), which confirms the presence of Ni^2+^ (Figure 3g). There are two peaks in Figure 3h, at approximately 711.79 eV and 725.48 eV, with satellite peaks at 717.09 eV and 733.69 eV, which are evidence of the presence of Fe^3+^ [50]. The above XPS results are in agreement with those reported in the literature, which further confirms the coexistence of CdS and NiFe-LDH in the NiFe-LDH/CdS/BiVO_4_ photoanode.

### 3.2. PEC Properties of the Photoanodes

**Figure 4:**
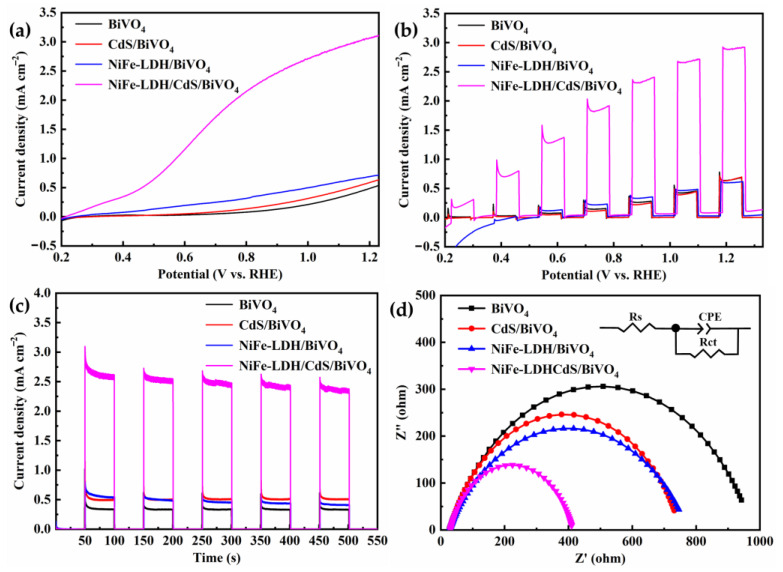
(**a**) Linear sweep voltammetry curves (LSV), (**b**) transient photocurrent curves measured under chopped light (on or off cycle: 8 s), (**c**) photocurrent–time curves measured at 1.23 V under chopped illumination, and (**d**) electrochemical impedance spectroscopy in a 0.5 M Na_2_SO_4_ electrolyte (pH = 6.1) at 1.23 V under illumination of the photoanodes.

The PEC performances of the photoanodes were evaluated by measuring the photocurrent density versus applied potential curves under AM 1.5 G solar illumination from a xenon lamp in a 0.5 M Na_2_SO_4_ electrolyte at pH 6.1. As demonstrated in Figure 4a, at 1.23 V, the photocurrent density of bare BiVO_4_ is 0.53 mA cm^−2^. In comparison, the CdS/BiVO_4_ and NiFe-LDH/BiVO_4_ photoanodes yield marginally higher values, attaining 0.63 mA cm^−2^ and 0.71 mA cm^−2^, respectively. Strikingly, the NiFe-LDH/CdS/BiVO_4_ photoanode delivers a photocurrent density of 3.1 mA cm^−2^, surpassing CdS/BiVO_4_ by 4.9-fold, NiFe-LDH/BiVO_4_ by 4.3-fold, and bare BiVO_4_ by 5.8-fold. Moreover, the onset potentials for CdS/BiVO_4_, NiFe-LDH/BiVO_4_, and NiFe-LDH/CdS/BiVO_4_ photoanodes exhibit cathodic shifts of 140 mV, 430 mV, and 540 mV, respectively, relative to BiVO_4_. These findings imply that a single-component modification of BiVO_4_ only with CdS or NiFe-LDH is barely adequate to improve the PEC performance. However, a significant enhancement of PEC performance could be achieved through dual modification with CdS and NiFe-LDH. The light/dark photocurrent response, depicted in Figure 4b, confirms the excellent optical switching behavior and high visible light sensitivity of all photoanodes. Among them, the NiFe-LDH/CdS/BiVO_4_ photoanode demonstrates the most pronounced photoresponse across the entire voltage range. Additionally, the current densities of the photoanodes display minimal decay, eventually stabilizing, as shown in Figure 4c, indicative of their good stability throughout the chronoamperometric tests.

The charge transfer capabilities of the photoanodes were assessed using electrochemical impedance spectroscopy (EIS) across a frequency range of 0.1 Hz to 100 kHz under illumination. The resulting Nyquist data were analyzed and fitted by an equivalent circuit model to interpret the impedance characteristics. In general, the semicircle diameter in an EIS Nyquist plot is indicative of the charge transfer resistance (R_ct_) at the photoanode/electrolyte interface; a smaller diameter corresponds to a reduced R_ct_, denoting enhanced charge transfer kinetics [51]. The NiFe-LDH/CdS/BiVO_4_ photoanode demonstrated a significantly lower R_ct_ of 420 Ω, as compared to 950 Ω for bare BiVO_4_, 740 Ω for CdS/BiVO_4_, and 760 Ω for NiFe-LDH/BiVO_4_, as detailed in Figure 4d and Table S2. This reduction in R_ct_ for the NiFe-LDH/CdS/BiVO_4_ photoanode suggests that the dual modification with CdS and NiFe-LDH synergistically diminishes charge transfer resistance, thereby facilitating OER at the interface [52].

**Figure 5:**
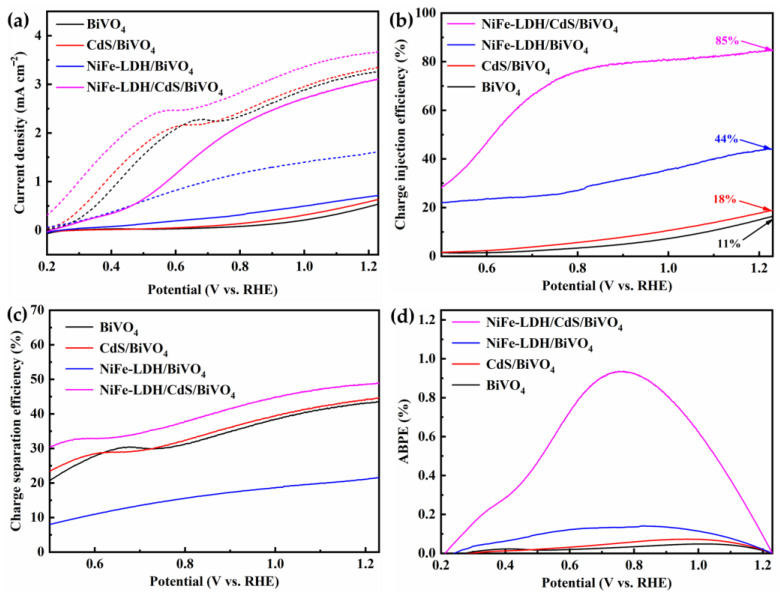
(**a**) LSV curves in 0.5 M Na_2_SO_4_ electrolyte (pH = 6.1) with (dashed line) and without (solid line) 0.5 M sodium sulfite; (**b**) charge injection and (**c**) charge separation efficiencies of the photoanodes in 0.5 M Na_2_SO_4_ (pH = 6.1) with AM 1.5 G-simulated sunlight at 100 mW cm^−2^; (**d**) applied bias photon-to-current efficiency for the photoanodes.

As the oxidation of sodium sulfite is more kinetically facile than the oxidation of water, the bulk properties of the prepared photoanodes were investigated in the presence of sodium sulfite to exclude the influence of the slow water oxidation kinetics [53]. Figure 5a illustrates the LSV curves obtained in a 0.5 M Na_2_SO_4_ (pH 6.1) electrolyte, both with and without the addition of 0.5 M sodium sulfite. Indeed, for all photoanodes, the photocurrent generated for sulfite oxidation was noticeably higher than that generated for water oxidation, in particular for bare BiVO_4_, whose photocurrent at 1.23 V is significantly higher with Na_2_SO_3_ compared to without, indicating that the photoconversion efficiency is severely hindered by slow OER kinetics. The charge injection efficiency (η_inj_), which describes the fraction of injected holes from the electrode surface into the electrolyte, was then calculated according to Equation (2), and the result is plotted in Figure 5b. The NiFe-LDH/CdS/BiVO_4_ photoanode has a η_inj_ of 85% at 1.23 V, which is much larger than that of BiVO_4_ (11%), CdS/BiVO_4_ (18%), and NiFe-LDH/BiVO_4_ (44%). Figure 5c illustrates the charge separation efficiency (η_sep_) of the photoanodes. An enhancement was also observed for the NiFe-LDH/CdS/BiVO_4_ photoanode, but it was less pronounced. 

**Figure 6:**
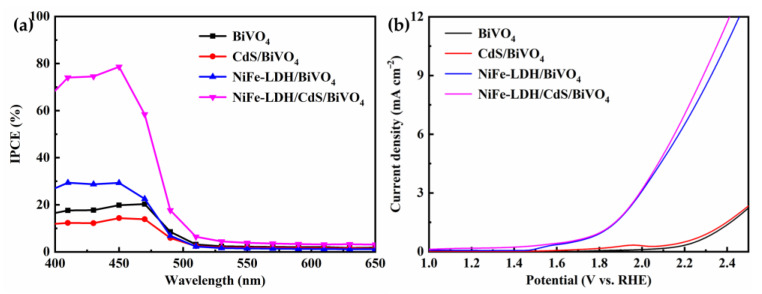
(**a**) IPCE curves obtained at 1.23 V under illumination; (**b**) polarization curves of the photoanodes measured in a 0.5 M Na_2_SO_4_ (pH = 6.1) solution in the dark.

The photoconversion efficiencies of the photoanodes were evaluated by the applied bias photon-to-current conversion efficiency (ABPE), calculated from the *J* − *V* curves according to Equation (3). As revealed in Figure 4d, the NiFe-LDH/CdS/BiVO_4_ photoanode possesses the highest ABPE of 0.93% at a lower potential (0.75 V), a significant improvement compared to BiVO_4_ (0.048% at 1.01 V), CdS/BiVO_4_ (0.073% at 0.96 V), and NiFe-LDH/BiVO_4_ (0.14% at 0.83 V). The incident photon-to-current efficiency (IPCE), another important index for evaluating photoconversion efficiencies, was further examined. The NiFe-LDH/CdS/BiVO_4_ photoanode achieved a superior IPCE of 78.6% at 450 nm, outperforming BiVO_4_ (20.3%), CdS/BiVO_4_ (14.3%), and NiFe-LDH/BiVO_4_ (29.3%) (Figure 6a).

To illustrate the important role of NiFe-LDH in the composite photoanodes, the OER electrocatalytic performance of the photoanodes was also investigated by polarization curves measured under dark conditions (Figure 6b). In contrast to BiVO_4_ and CdS/BiVO_4_, which show minimal OER activity, the NiFe-LDH/BiVO_4_ and NiFe-LDH/CdS/BiVO_4_ photoanodes exhibit markedly increased water oxidation currents, confirming the OER enhancement by NiFe-LDH. All the above outcomes demonstrate that the integration of CdS and NiFe-LDH advances photogenerated charge separation and transfer while also accelerating water oxidation kinetics, synergistically boosting the PEC water splitting performance. Notably, the PEC parameters of the NiFe-LDH/CdS/BiVO_4_ photoanode are comparable with, or surpass, several recently documented BiVO_4_-based photoanodes, as compiled in Table S3.

To examine the influence of carrier density on the photoelectrochemical performance, Mott−Schottky (M−S) analysis was conducted for BiVO_4_, CdS, NiFe-LDH, and their composites at a frequency of 1 kHz in dark conditions, as presented in Figure S3. The M−S plots consistently exhibited positive slopes, confirming the n-type semiconductor nature of the materials. The flat band potentials (E_fb_) were determined for BiVO_4_, CdS, NiFe-LDH, CdS/BiVO_4_, NiFe-LDH/BiVO_4_, and NiFe-LDH/CdS/BiVO_4_ by extrapolation of their M−S plots, and the values are tabulated in Table S4. The more negative E_fb_ of CdS, relative to BiVO_4_, indicates that the photoexcited electrons in CdS can transfer to the conducting band of BiVO_4_, while the holes of BiVO_4_ transfer to the valence band of CdS, thus suppressing the recombination of photogenerated electron–hole pairs [43]. Carrier densities (N_D_) of the photoanodes were also deduced from the slop of the M−S curves and are also listed in Table S4. The NiFe-LDH/CdS/BiVO_4_ photoanode exhibited the highest N_D_ value, which is about twice compared to the CdS/BiVO_4_ or NiFe-LDH/BiVO_4_ photoanode, again verifying more efficient charge separation and hole injection after dual modification.

The photostability of the photoanodes was tested by subjecting them to continuous illumination at 1.23 V for 3 h in a 0.5 M Na_2_SO_4_ solution. In contrast to CdS/BiVO_4_, whose photocurrent density drastically fell to a mere 14% of its initial value over this period, NiFe-LDH/CdS/BiVO_4_ maintained nearly 50% of its initial photocurrent density under identical conditions (Figure S4). This endurance suggests that the NiFe-LDH nanosheet layer functions effectively as a hole shuttle to transfer holes accumulated at the CdS/BiVO_4_ interface under the applied potential and, at the same time, protects CdS from photocorrosion. Simultaneously, Ni^2+^ ions within NiFe-LDH undergo oxidation by holes from the CdS valence band to higher valence species, which oxidize hydroxide ions (OH^−^) to produce O_2_ gas and then return to their original valence state. Meanwhile, electrons are transferred to the counter electrode (Pt) via the external circuit and reduce protons (H^+^) into H_2_ gas (Figure S5). The generation of H_2_ and O_2_ gases by the NiFe-LDH/CdS/BiVO_4_ photoanode was verified and quantified using gas chromatography (GC) integration (Figure S6). After 3 h of operation, the measured volumes of H_2_ and O_2_ evolution were 131 µmol and 65 µmol, respectively.

## 4. Conclusions

This work reports the frabrication of a triadic photoanode NiFe-LDH/CdS/BiVO_4_ by co-modification of BiVO_4_ with CdS nanoparticles and NiFe-LDH nanosheets. The n-n heterojunction created at the interface of CdS and BiVO_4_ significantly improves charge separation and transfer through alleviating charge carrier recombination. The presence of NiFe-LDH further augments this effect by boosting the kinetics of hole transfer from the CdS/BiVO_4_ junction. This synergistic effect of CdS and NiFe-LDH thus markedly enhances the charge injection and separation efficiencies, ABPE, and IPCE values for the NiFe-LDH/CdS/BiVO_4_ photoanode relative to the singular BiVO_4_ and the binary CdS/BiVO_4_ and NiFe-LDH/BiVO_4_ counterparts. As a result, the photocurrent density under illumination climbs from 0.53 mA cm^−2^ for pristine BiVO_4_ to 3.1 mA cm^−2^ for the NiFe-LDH/CdS/BiVO_4_ photoanode, while the onset potential decreases from 920 mV to 380 mV, indicating a cathodic shift of 540 mV. In addition, the NiFe-LDH/CdS/BiVO_4_ photoanode demonstrates considerable stability with sustained photocurrent density over 3 h of irradiation. These findings pave the way for the development of effective and sustainable non-precious metal photoelectrocatalysts for PEC water splitting technologies.

## Data Availability

Data is contained within the article.

## Supplementary Materials

The following supporting information can be downloaded at: https://www.mdpi.com/article/10.3390/nano14131100/s1, Figure S1: SEM images of (a) BiOI and (b) CdS/BiVO_4_. Figure S2: (a) EDS and (b) mapping of NiFe-LDH/CdS/BiVO_4_. Figure S3: M−S plots of (a) NiFe-LDH, (b) CdS, and (c) the photoanodes measured in a 0.5 M Na_2_SO_4_ solution (pH = 6.1) in the dark. Figure S4: Stability testing of the photoanodes at 1.23 V under illumination. Figure S5: Schematic illustration of the PEC water oxidation for the NiFe-LDH/CdS/BiVO_4_ photoanode. Figure S6: H_2_ and O_2_ gas evolution using the NiFe-LDH/CdS/BiVO_4_ photoanode; Table S1: SEM-EDS mapping elemental analysis of NiFe-LDH/CdS/BiVO_4_. Table S2: The fitted results of EIS data using an equivalent circuit model. Table S3: The recent literature summary of BiVO_4_-based photoanodes at 1.23 V under AM 1.5 G illumination (100 mW cm^−2^). Table S4: Carrier density and flat band potentials of the photoanodes. References [54,55,56,57,58,59,60,61,62,63,64,65] are cited in the supplementary materials.
