# Supplementary material for: Promoting the Photoelectrochemical Properties of BiVO4 Photoanode via Dual Modification with CdS Nanoparticles and NiFe-LDH Nanosheets
Source: Nanomaterials (Basel). 2024 Jun 26;14(13):1100. doi: 10.3390/nano14131100 (PMC11242967; doi:10.3390/nano14131100)
Supplement: Supplementary file 1 [file nanomaterials-14-01100-s001.zip › nanomaterials-3010457-supplementary.pdf]

Supporting Information

Promoting Photoelectrochemical Properties of $BiVO_4$ Photoanode via Dual Modification with CdS Nanoparticles and NiFe-LDH Nanosheets

Guofa Dong [1], Tingting Chen [1], Fangxia Kou [1], Fengyan Xie [1], Caihong Xiao [1], Jiaqi Liang [2], Chenfang Lou [2], Jiandong Zhuang [2] and Shaowu Du [1,*]

1 Fuzhou Institute of Oceanography, College of Materials and Chemical Engineering, Minjiang University, Fuzhou 350108, China; swdu@mju.edu.cn (S. D.); gfdong@mju.edu.cn (G. D.)

2 College of Materials Engineering, Fujian Agriculture and Forestry University, Fuzhou 350002, China

* Correspondence: swdu@mju.edu.cn (S. D.)

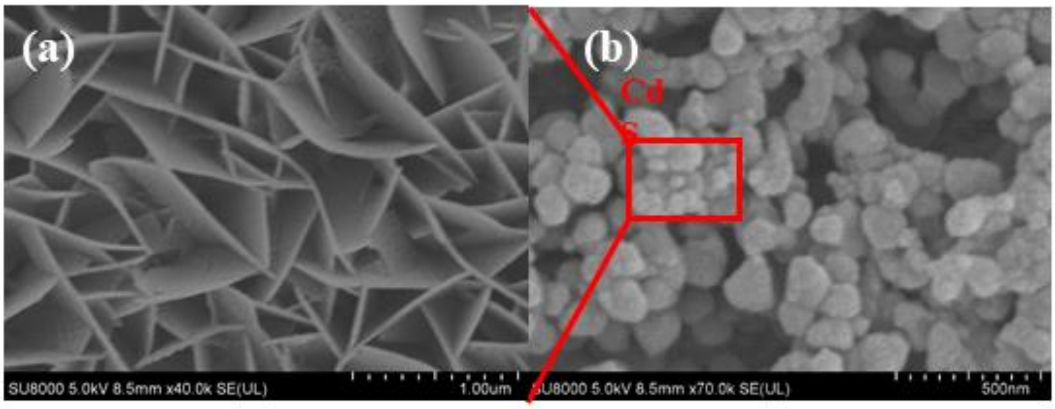

Figure S1. SEM images of (a) BiOI, and (b) $CdS/BiVO_4$.

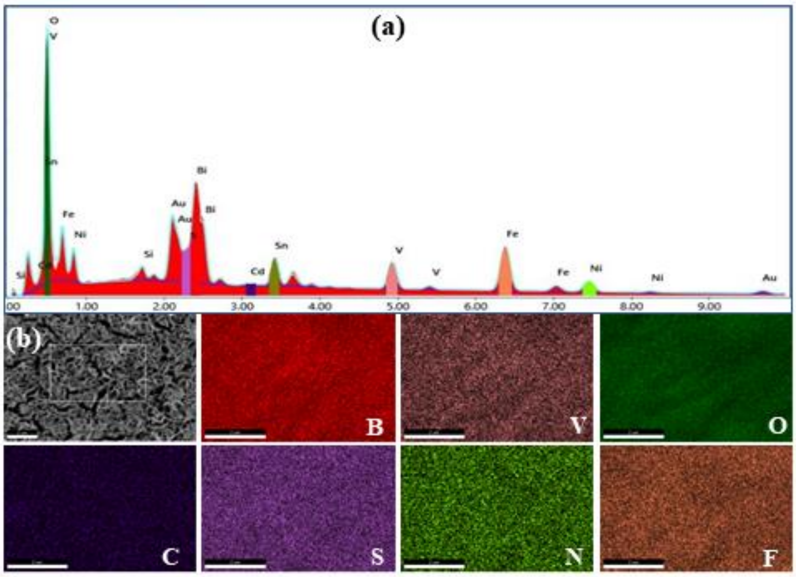

Figure S2. (a) EDS and (b) mapping of $NiFe-LDH/CdS/BiVO_4$.

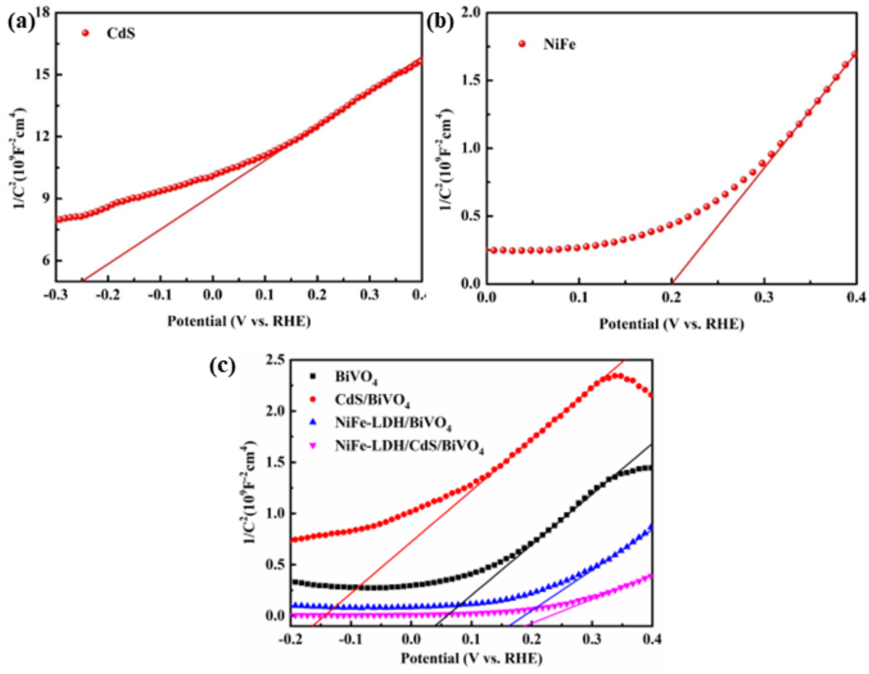

Figure S3. M−S plots of (a) NiFe-LDH, (b) CdS and (c) the photoanodes measured in a 0.5 M Na_2SO_4 solution (pH = 6.1) in dark.

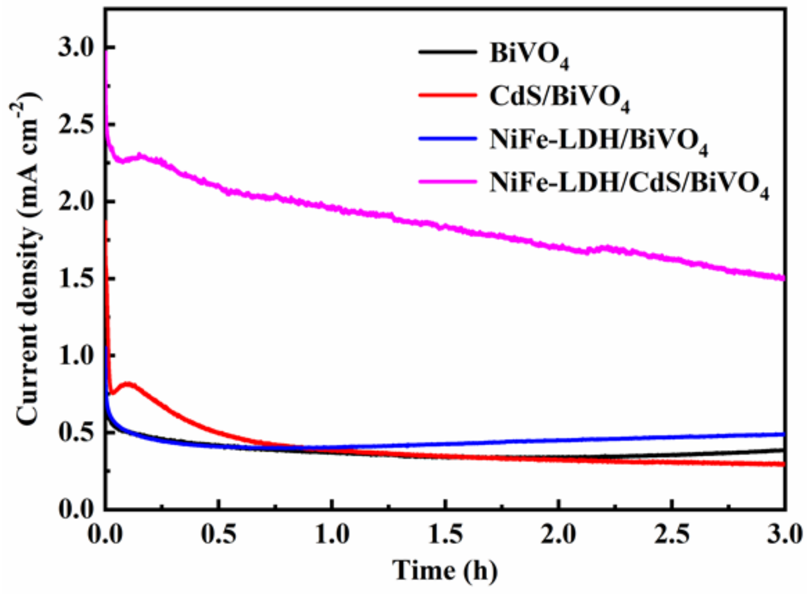

Figure S4. Stability testing of the photoanodes at 1.23 V under illumination.

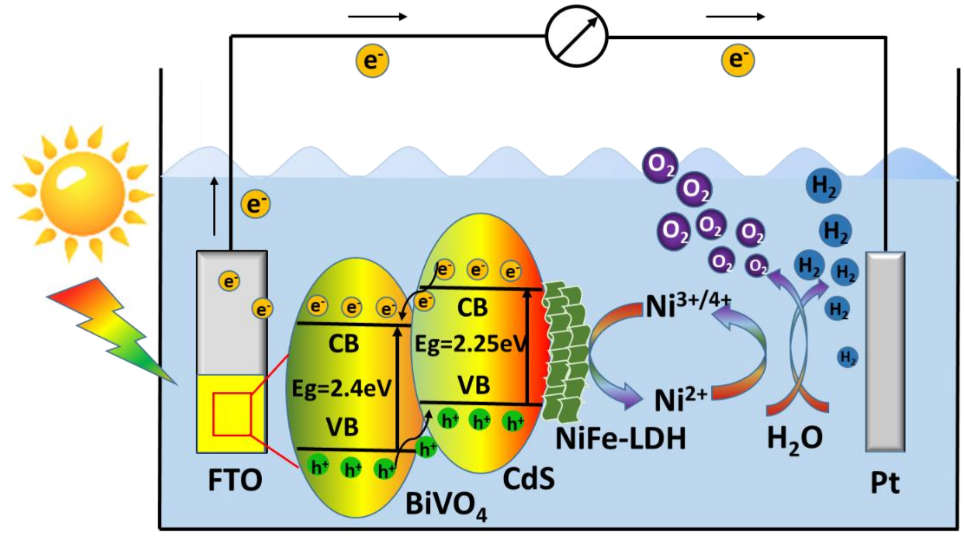

Figure S5. Schematic illustration of the the PEC water oxidation for the NiFe-LDH/CdS/BiVO$_4$ photoanode.

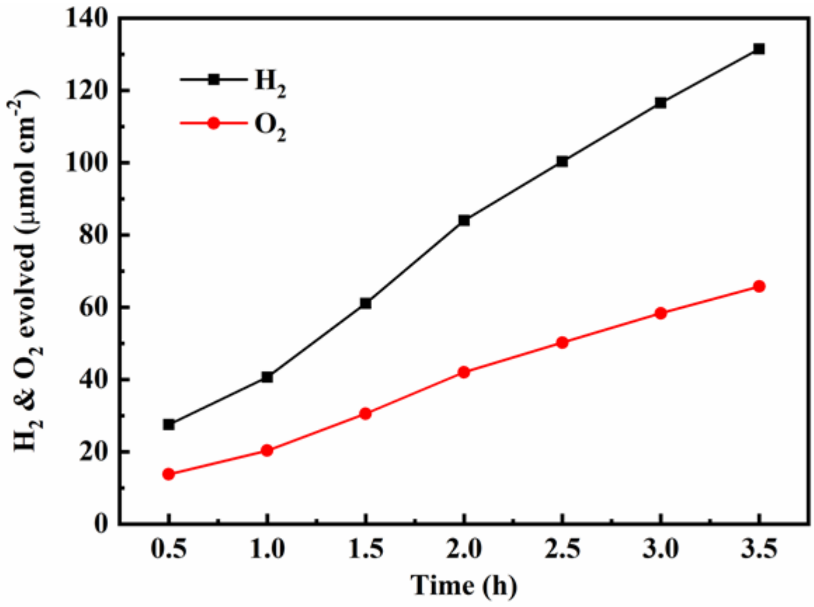

Figure S6. H_2 and O_2 gases evolution using the NiFe-LDH/CdS/BiVO$_4$ photoanode.

Table S1. SEM-EDS mapping elemental analysis of NiFe-LDH/CdS/$BiVO_4$.

Element	Weight %	Atomic %
O K	27.3	62.5
V K	7.6	5.5
Fe K	21.1	14.2
Ni K	7.2	4.7
S K	4.7	5.3
Cd L	2.3	1.8
Sn L	9.5	2.8
Bi M	20.3	3.2

Table S2. The fitted results of EIS data using an equivalent circuit model.

Photoanodes	R_s (Ω)	R_{ct} (Ω)
$BiVO_4$	27	950
CdS/$BiVO_4$	29	740
NiFe-LDH/$BiVO_4$	27	760
NiFe-LDH/CdS/$BiVO_4$	30	420

Table S3. The recent literature summary of $BiVO_4$-based photoanodes at 1.23 V *vs.* RHE under AM 1.5 G illumination (100 mW cm^{-2}).

Photoanodes	Electrolyte	J_{ph} mA cm^{-2}	η_{inj}	ABPE	IPCE	Ref.
$BiVO_4$/ZnCo-MOF	0.5 M Na_2SO_4	3.08	/	0.5%	/	[1]
$BiVO_4$@Ni:FeOOH	0.5 M Na_2SO_4	2.86	90.2%	/	78%	[2]
$BiVO_4$/Zn-MOF	0.5 M Na_2SO_4	1.46	76.8%	0.36%	28.8%	[3]
F:FeOOH/$BiVO_4$	0.5 M Na_2SO_4	2.70	56.0%	0.66%	50%	[4]
CQDs/FeOOH/$BiVO_4$	0.2 M Na_2SO_4	2.53	81.1%	0.60%	42.6%	[5]
NiCo-LDH/$BiVO_4$	0.5 M Na_2SO_4	3.40	71.0%	0.66%	59%	[6]
H-CoAl-LDH/$BiVO_4$	0.5 M Na_2SO_4	3.50	78.0%	0.85%	57.1%	[7]
β-FeOOH/$BiVO_4$	0.2 M Na_2SO_4	4.30	80.0%	0.71%	55%	[8]
NiO/$BiVO_4$	0.5 M Na_2SO_4	1.94	64%	0.37%	31.7%	[9]
$BiVO_4$/NiFeOOH/Co-Pi	0.5 M Na_2SO_4	2.03	/	0.44%	53.25%	[10]
$Ni_xCo_{1-x}O$/$BiVO_4$	0.5 M Na_2SO_4	3.2	59%	0.75%	42.5%	[11]
$BiVO_4$-MOF-N_2	0.5 M Na_2SO_4	2.32	63.7%	/	/	[12]
CoS/$BiVO_4$	0.5 M PBS	3.20	75.9%	/	70.6%	[13]
NiFe-LDH/CdS/$BiVO_4$	0.5 M Na_2SO_4	3.10	85%	0.93	78.6%	This work

Table S4. Carrier density and flat band potentials of the photoanodes.

Samples	N_D (cm^{-3})	E_{fb} (V)
$BiVO_4$	2.86×10^{22}	0.04
CdS	8.46×10^{21}	−0.25
NiFe	1.66×10^{22}	0.2
CdS/$BiVO_4$	2.81×10^{22}	−0.16
NiFe-LDH/$BiVO_4$	3.58×10^{22}	0.16
NiFe-LDH/CdS/$BiVO_4$	6.04×10^{22}	0.18

References

[1] Kubendhiran, S.; Chung, R.-J.; Yougbaré, S.; Lin, L.-Y.; Wu, Y.-F. Enhanced Photoelectrochemical Water Oxidation on $BiVO_4$ by Addition of ZnCo-MOFs As Effective Hole Transfer Co-Catalyst. *Int. J. Hydrogen. Energ.* **2023**, *48*, 101–112.

[2] Zhang, X.; Li, H.; Kong, W.; Liu, H.; Fan, H.; Wang, M. Reducing the Surface Recombination During Light-Driven Water Oxidation by Core-Shell $BiVO_4$@Ni:FeOOH. *Electrochim. Acta* **2019**, *300*, 77–84.

[3] Bai, H.; Wang, F.; You, Z.; Sun, D.; Cui, J.; Fan, W. Fabrication of Zn-MOF Decorated $BiVO_4$ Photoanode for Water Splitting. *Colloid Surface A* **2022**, *640*, 128412.

[4] She, H.; Yue, P.; Huang, J.; Wang, L.; Wang, Q. One-Step Hydrothermal Deposition of F:FeOOH onto $BiVO_4$ Photoanode for Enhanced Water Oxidation. *Chem. Eng. J.* **2020**, *392*, 123703.

[5] Zhou, T.; Chen, S.; Wang, J.; Zhang, Y.; Li, J.; Bai, J.; Zhou, B. Dramatically Enhanced Solar-Driven Water Splitting of $BiVO_4$ Photoanode via Strengthening Hole Transfer and Light Harvesting by Co-Modification of CQDs and Ultrathin β-FeOOH Layers. *Chem. Eng. J.* **2021**, *403*, 126350.

[6] She, H.; Yue, P.; Ma, X.; Huang, J.; Wang, L.; Wang, Q. Fabrication of $BiVO_4$ Photoanode Ccatalyzed with NiCo-layered Duble Hdroxide for Ehanced Potoactivity of Water Oxidation. *Appl. Catal. B: Environ.* **2020**, *263*, 118280.

[7] Yue, P.; She, H.; Zhang, L.; Niu, B.; Lian, R.; Huang, J.; Wang, L. Super-Hydrophilic CoAl-LDH on $BiVO_4$ for Enhanced Photoelectrochemical Water Oxidation Activity. *Appl. Catal. B: Environ.* **2021**, *286*, 119875.

[8] Zhang, B.; Wang, L.; Zhang, Y.; Ding, Y.; Bi, Y. Ultrathin FeOOH Nanolayers with Abundant Oxygen Vacancies on $BiVO_4$ Photoanodes for Efficient Water Oxidation. *Angew. Chem. Int. Ed.* **2018**, *57*, 2248–2252.

[9] Zhang, S.; Lu, Y.; Ding, Q.; Yu, Y.; Huo, P.; Shi, W.; Xu, D. MOF Derived NiO Thin Film Formed p-n Heterojunction with $BiVO_4$ Photoelectrode for Enhancement of PEC Performance. *Colloid Surface A* **2022**, *655*, 130282.

[10] Fang, G.; Liu, Z.; Han, C. Enhancing the PEC Water Splitting Performance of $BiVO_4$ Co-Modifying with NiFeOOH and Co-Pi Double Layer Cocatalysts. *Appl. Surf. Sci.* **2020**, *515*, 146095.

[11] Huang, J.; Luo, W.; Yuan, X.; Wang, J. Bimetallic MOF-Derived Oxides Modified $BiVO_4$ for Enhanced Photoelectrochemical Water Oxidation Performance. *J. Alloy. Compd.* **2023**, *930*, 167397.

[12] Wang, D.; Gu, J.; Wang, H.; Liu, M.; Liu, Y.; Zhang, X. Promoting Photoelectrochemical Water Oxidation of $BiVO_4$ Photoanode via Co-MOF-Derived Heterostructural Cocatalyst, *Appl. Surf. Sci.* **2023**, *619*, 156710.

[13] Zhou, Z.; Chen, J.; Wang, Q.; Jiang, X.; Shen, Y. Enhanced Photoelectrochemical Water Splitting Using a Cobalt-Sulfide-Decorated $BiVO_4$ Photoanode. *Chinese J. Catal.* **2022**, *43*, 433–441.
